# Hydrogenated Amorphous TiO_2−x_ and Its High Visible Light Photoactivity

**DOI:** 10.3390/nano11112801

**Published:** 2021-10-22

**Authors:** Guang Feng, Mengyun Hu, Shuai Yuan, Junyi Nan, Heping Zeng

**Affiliations:** 1 Shanghai Key Laboratory of Modern Optical System, Engineering Research Center of Optical Instrument and System, Ministry of Education, School of Optical-Electrical and Computer Engineering, University of Shanghai for Science and Technology, Shanghai 200093, China; sunnyfeng1992@163.com (G.F.); myhu@phy.ecnu.edu.cn (M.H.); ye_zoom@126.com (S.Y.); 2 Chongqing Key Laboratory of Precision Optics, Chongqing Institute of East China Normal University, Chongqing 401120, China; 3 State Key Laboratory of Precision Spectroscopy, East China Normal University, Shanghai 200062, China; nanjunyigood@163.com; 4 CAS Center for Excellence in Ultra-Intense Laser Science, Shanghai 201800, China; 5 Jinan Institute of Quantum Technology, Jinan 250101, China

**Keywords:** hydrogenated TiO_2−x_, oxygen vacancy, hydrogenation, liquid plasma, band structure

## Abstract

Hydrogenated crystalline TiO_2_ with oxygen vacancy (O_V_) defect has been broadly investigated in recent years. Different from crystalline TiO_2_, hydrogenated amorphous TiO_2−x_ for advanced photocatalytic applications is scarcely reported. In this work, we prepared hydrogenated amorphous TiO_2−x_ (HA-TiO_2−x_) using a unique liquid plasma hydrogenation strategy, and demonstrated its highly visible-light photoactivity. Density functional theory combined with comprehensive analyses was to gain fundamental understanding of the correlation among the O_V_ concentration, electronic band structure, photon capturing, reactive oxygen species (ROS) generation, and photocatalytic activity. One important finding was that the narrower the bandgap HA-TiO_2−x_ possessed, the higher photocatalytic efficiency it exhibited. Given the narrow bandgap and extraordinary visible-light absorption, HA-TiO_2−x_ showed excellent visible-light photodegradation in rhodamine B (98.7%), methylene blue (99.85%), and theophylline (99.87) within two hours, as well as long-term stability. The total organic carbon (TOC) removal rates of rhodamine B, methylene blue, and theophylline were measured to 55%, 61.8%, and 50.7%, respectively, which indicated that HA-TiO_2−x_ exhibited high wastewater purification performance. This study provided a direct and effective hydrogenation method to produce reduced amorphous TiO_2−x_ which has great potential in practical environmental remediation.

## 1. Introduction

Hydrogenated crystalline TiO_2−x_ (C-TiO_2−x_) has been extensively investigated owing to its full-spectra absorption and effective solar energy conversion, deriving from the self-doped states created by O_V_ and Ti^3+^ species [1,2,3]. Apart from crystalline TiO_2_, amorphous TiO_2_ as a common type of titanium oxide has not been reported on after hydrogenation for its photoactivity utilization. Unlike C-TiO_2−x_, amorphous TiO_2_ typically possesses many special properties including characteristic long-range disordered structure, high specific surface area, and narrow bandgap [4,5,6,7,8]. In order to tailor more narrower bandgap and acquire more photons utilization, amorphous TiO_2_ should be considered as an ideal candidate for hydrogenation treatments, which could largely boost the photoactivity and bring about some original and significant physicochemical observations. Unfortunately, owing to its poor solar energy conversion and ineffective charge separation, amorphous TiO_2_ is ignored as an advanced photocatalyst [9,10,11]. More importantly, hydrogenation with amorphous TiO_2_ inevitably calls for annealing or thermal hydrogenation, which enables its crystallization and totally changes into hydrogenated anatase/rutile TiO_2−x_. Herein, it is a huge challenge to synthesize hydrogenated amorphous TiO_2−x_ (HA-TiO_2−x_) and, particularly, equip it with the oxygen vacant disordered surface.

In addition, there arises a controversy on the origins of low-energy photon absorption and band structure regulation theory in hydrogenated TiO_2−x_ nanomaterials [12,13,14]. Some viewpoints consider that the disordered surface layer, instead of the crystalline core, is responsible for the low-energy photons absorption [15,16]. But others suggest both disordered surface and crystalline core, as well as the interface, play a synergistic effect on capability of capturing visible to infrared light [17,18]. To clarify the contribution of disordered surface and crystalline core, constructing a distinctive model of a disordered surface with an amorphous core can exclude the influences of a crystalline core, and could lead to more explicit investigations on its photoactivity mechanism. Considering these aspects, the aim of this work is to produce HA-TiO_2−x_ with disordered surface with an amorphous core configuration, which may reveal unusual electrical and optical properties deviated from traditional hydrogenated TiO_2−x_.

**Figure 1:**
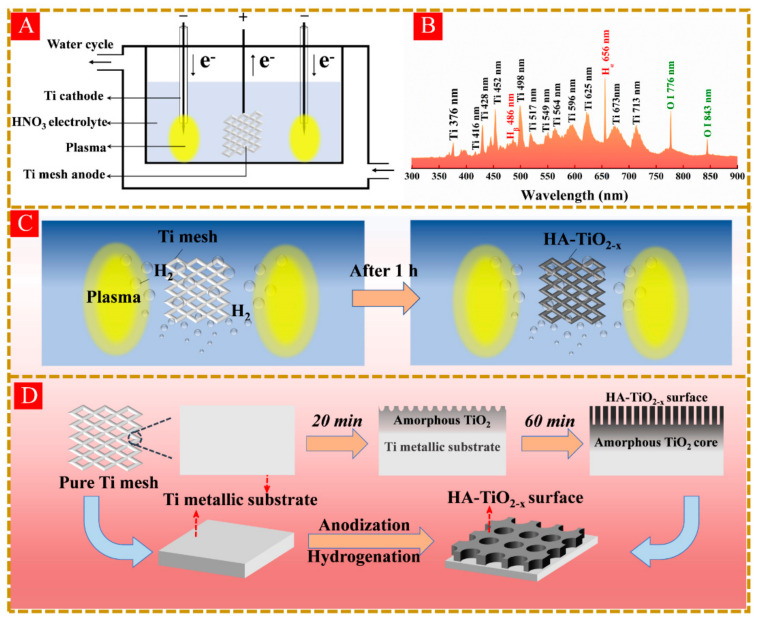
(**A**) the setup of synergistic treatment of liquid plasma induced hydrogenation and anodization. (**B**) the emission spectra of liquid plasma, and (**C**) the variations of surface color in Ti mesh. (**D**) schematic illustration of the preparation of HA-TiO_2−x_@Ti mesh photocatalysts.

In this work, we conducted an in-situ synthesis of HA-TiO_2−x_ using a synergistic method involving anodization and liquid-plasma induced hydrogenation. As shown in Figure 1A, the synthesis setup was composed of one titanium mesh anode, two titanium rod cathodes, and electrolytic cell. Once applied high-voltage pulses, anodization reaction occurred and resulted in the generation of nanopores on the surface of Ti mesh. Meanwhile, two bright liquid plasma were generated on the surfaces of cathodic titanium rods. The optical emission spectrum of liquid plasma as shown in Figure 1B, which clearly exhibited various emission peaks including Ti I (neutral), Ti II (single-charged ions), hydroxyl radicals, hydrogen, and atomic oxygen. A distinguished hydrogen atom peak at 656 nm confirmed the production of massive hydrogen atoms associated with the hydrogen reduction environment in electrolyte [19,20]. As described above, the Ti mesh thus experienced in-situ synergistic treatments including anodization and liquid-plasma induced hydrogenation. As shown in Figure 1C, after 1 h synergistic treatments, the color of Ti mesh changed from silvery white to dark gray, along with lots of nanopores emerging on the surface shown in Figure 1D. We hereafter dubbed the sample according to the treatment time, for example AT-60 refers to the HA-TiO_2−x_ obtained by applying a treatment time of 60 min.

This is the first report on the synthesis of hydrogenated amorphous TiO_2−x_ to the best of our knowledge. Behind the systematic optical and electrical investigations, HA-TiO_2−x_ was found to exhibit superior visible light photoactivity as well as long-term stability. Based on the comprehensive experimental and theoretical analyses including electron paramagnetic resonance, X-ray photoelectron spectroscopy, positron annihilation spectrometry, and density functional theory results, the correlation among O_V_ concentration, electrical structure, optical property, and photoactivity were clarified. The shallow states bellow the conduction band and above the valence band were formed in HA-TiO_2−x_, which originated from the oxygen vacant disordered surface. The extended shallow states between the bandgap was responsible for the extraordinary visible-light absorption. With the benefits of excellent visible-light absorption and robust surface O_V_, HA-TiO_2−x_ exhibited superior and stable visible-light photodegradations, but alternatively, poor photoactivity in the UV region. After analysing the reactive oxygen species (ROS) and its scavenging experiments, uncommon occurrence was observed that electrons were hardly not transferred from the valence band to O_V_ induced shallow states or conduction band, thus preventing the generation of h^+^ radicals and reducing the UV-responded photoactivity. The synthesis of HA-TiO_2−x_ avoided the annealing and crystallization processes, simplified its preparation procedures, saved the costs, and reduced the consumption, which most likely lead to breakthroughs in nano-architecture of novel amorphous photocatalysts for practical and industrial applications.

## 2. Experimental Section

### 2.1. Reagents and Materials

Ti mesh (purity 99%) was purchased from Hebei Borui Metal Materials Co., Ltd. (Handan, China). All chemicals with analytical grade and no further purification were purchased from Shanghai Aladdin Biochemical Technology Co., Ltd. (Shanghai, China).

### 2.2. Preparation of HA-TiO_2−x_

One anodic titanium mesh (20 × 20 × 1 mm^3^, purity 99%) and two cathodic titanium rods (4 mm diameter, purity 99%) sealed into a corundum tube were placed in a cell filled with 300 mL nitric acid electrolyte (HNO_3_) as shown in Figure 1A. Two cathodes were used to generate glow discharges and avoid unbalanced flow and temperature gradients in the HNO_3_ electrolyte. Pulsed voltages were applied between anode and cathodes to produce intense plasma on the cathode surfaces. Liquid plasma was produced with an appropriate pulse voltage power (600 V, 1 kHz). To prevent the electrolyte evaporation, a water chiller was used to maintain the electrolyte temperature at 80 °C. After the synergy treatments, HA-TiO_2−x_ was washed with ultrasonic wave and dried with oven at 50 °C for 12 h. All samples were treated with the same output power of 420 W (600 V, 0.7 A, 1 kHz). Hereafter, we dub the sample according to the treatment time, for example AT-60 refers to the HA-TiO_2−x_ obtained by applying a treatment time with 60 min.

The amorphous TiO_2_ nanopowder was synthesized by hydrolysis of tetrabutyl titanate under ambient conditions. Firstly, 2 mL tetrabutyl titanate and 5 mL deionized water were mixed for 1 h, and then the mixture was dried with oven under 30 °C for 10 h. The white amorphous TiO_2_ nanopowder was then prepared.

### 2.3. Characterization

The phase and crystallinity for all samples were tested by X-ray powder diffraction (XRD) using a Rigaku Smartlab (Rigaku) machine equipped with Cu Ka irradiation (λ = 1.54056 Å). The morphology was characterized by scanning electron microscope (SEM) using a ZEISS MERLIN instrument operated at an acceleration voltage of 200 kV. High-resolution transmission electron microscopy (HRTEM) images were acquired using a JEOL JEM-2100F. UV-Vis diffused reflectance spectra (DRS) were measured by Shimadzu UV-2700 spectrophotometer at a wavelength range of 200–800 nm at room temperature. The X-ray photoelectron spectra (XPS) were recorded with thermos Escalab 250Xi. The existence of defects doped in the a-TiO_2−x_ nanoparticles was confirmed by the X-band electron paramagnetic resonance (EPR) spectra recorded at room temperature. The surface wettability of as-prepared sample was tested by angle-of-contact method using KINO SL200KB contact angle meter. The total organic carbon (TOC) of the reaction solution was determined using a Shimadzu TOC-L TOC analyzer.

The positron annihilation lifetime spectrum (PAS) used a ^22^Na positron emission source with an activity of about 2 × 10^6^ Bq. When it underwent β^+^ decay, it mainly produced positrons with kinetic energy of 0–540 keV and almost simultaneously emitted γ photons with energy of 1.28 MeV. Therefore, the appearance of this gamma photon can be regarded as the time starting point for the generation of positrons, and the appearance of 0.511 MeV annihilation gamma photons was the end of the positron annihilation event. This interval can be regarded as the lifetime of the positron. The radioactive source was sandwiched between the sample to form a sandwich structure with a total of 2 million counts, and the positron annihilation lifetime spectrum of the sample thus can be obtained. The time resolution of the system was about 190 picosecond (ps) and the track width was 12.5 ps.

### 2.4. Photodegradation Performances of HA-TiO_2−x_

The evaluation of visible-light photoactivity was tested with three typical wastewater pollutants, including rhodamine B (RhB), methyl blue (MB) and theophylline. We used a 300 W Xenon lamp with a 420 nm cut-off filter as the visible light source. The concentrations of RhB, MB and theophylline were all 10 mg/L. Black tea water pollutant was produced by 20 mg dry tea leaves that were put into 100 mL boiling water and cooled to room temperature, and the brown water was obtained when removal of tea leaves. Firstly, one HA-TiO_2−x_@Ti mesh (20 × 20 × 1 mm^3^) and 50 mL pollutant solution were put in a 500 mL beaker. Before illumination, the solution was placed in dark environment for 30 min with magnetic stirring for adsorption-desorption equilibrium. During photodegradation, we took 1 mL solution from the beaker with a fixed interval to analyse the time-dependent concentration of pollutant solution at specific wavelength by UV-2700 spectrophotometer, where RhB, MB, and theophylline were located at 554, 661, and 271.6 nm, respectively. Photodegradation experiments for all tested samples were carried out under the same conditions. In addition, the concentration of all sacrifice agents including ammonium oxalate (AO, h^+^ scavenger), Fe(II)-EDTA (H_2_O_2_ scavenger), potassium iodide (KI, OH_ads_ and electron scavenger), *p*-benzoquinone (BQ, O_2_^−^ scavenger), and isopropanol (IPA, scavenger for OH in the bulk solution) were 0.2 mM/mL. The EPR signals of radical spin-trapped by 5,5-dimethyl-1-pyrrolin-Noxide (DMPO) were recorded with visible-light illumination. In UV light mediated photodegradation, we used a 300 W Xenon lamp with a 365 nm cut-off filter as the UV light source (200–365 nm), and other experimental conditions including concentration of dyes and area of Ti mesh were unchanged.

The intermediate products of theophylline degradation were detected using high-performance liquid chromatography (HPLC) (Agilent 1290UPLC) and mass spectrometry (Agilent QTOF6550). The mobile phase consisted of 95% formic acid (0.1% concentration) and 5% acetonitrile at a constant flow rate of 0.3 mL/min. The injection volume was 2 μL. Separation was accompanied by using BEH C18 (1.7 μm, 2.1 × 50 mm) analytical column (Waters^®^ Acquity UPLC).

### 2.5. Theoretical Calculation Methods

We employed the Vienna Ab Initio Package (VASP) to perform all the density functional theory (DFT) calculations within the generalized gradient approximation (GGA) using the Perdew–Burke–Ernzerhof (PBE) formulation [21,22,23]. We selected the projected augmented wave (PAW) potentials to describe the ionic cores and take valence electrons into account using a plane wave basis set with a kinetic energy cutoff of 450 eV [24,25,26]. Partial occupancies of the Kohn–Sham orbitals were allowed using the Gaussian smearing method and a width of 0.05 eV. The electronic energy was considered self-consistent when the energy change was smaller than 10^−4^ eV. A geometry optimization was considered convergent when the force change was smaller than 0.05 eV/Å. Grimme’s DFT-D3 methodology was used to describe the dispersion interactions. We calculated the hydrogenation process on amorphous TiO_2_ surface by ab initio first-principles calculations with 10 ps.

## 3. Results and Discussion

### 3.1. Characterizations of HA-TiO_2−x_

**Figure 2:**
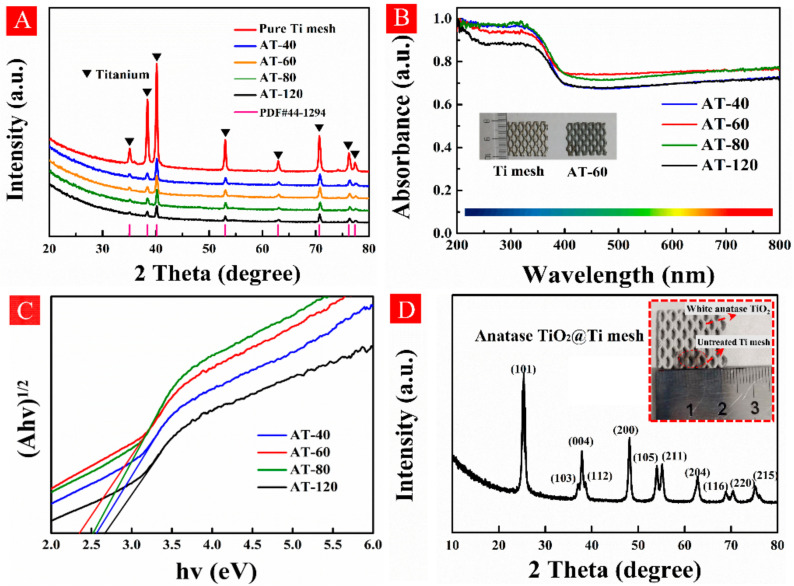
(**A**) X-ray diffraction (XRD) patterns and (**B**) diffuse reflectance spectroscopy of untreated Ti mesh and all samples. (**C**) the plots (αhν)^1/2^ versus hν by using the Kubelka–Munk function. (**D**) the XRD pattern of AT-60 sample after annealing treatments, and its color changed from gray to white (inset).

The crystal structures for all samples were detected by XRD analysis as shown in Figure 2A. The XRD patterns of all samples were nearly identical in characteristic peaks at 35.1°, 38.4°, 40.1°, 53°, 62.9°, 70.6°, 76.2°, and 77.3°, indexing to the (100), (002), (101), (102), (110), (103), (112), and (201) crystal faces of titanium metal (PDF#. 44-1294), respectively [27]. Obviously, the intensity of main peak at (101) facet decreased gradually with treatment time from 40 min to 120 min (i.e., from AT-40 to AT-120). Generally, the weakened intensity in diffraction peaks can be explained by long-range lattice disorder, which verified that amorphous TiO_2_ was generated on the Ti mesh surface, and in particular its concentration increased with the treatment time.

The DRS spectrum was used to evaluate the light absorption performances of all samples. As displayed in Figure 2B, Ti mesh showed almost no light absorption while all as-prepared samples exhibited a wide-range absorption from ultraviolet to visible and even infrared regions. Compared with silver color of Ti mesh, AT-60 showed dark grey color as displayed in the inset of Figure 2B. All treated samples followed the plots of (αhν)^1/2^ versus hν by using the Kubelka-Munk function [28], and from which the calculated bandgap of AT-40, AT-60, AT-80 and AT-120 were 2.57, 2.35, 2.52, and 2.66 eV, respectively, shown in Figure 2C. The decreased bandgap in our case implied some localized states caused by surface lattice defects could be created, for instance O_V_ and/or Ti^3+^ species [29]. To prove this assumption, AT-60 was heated at 400 °C for 3 h in the atmosphere and its XRD pattern in Figure 2D confirmed that anatase TiO_2_@Ti mesh was obtained. The color as expected changed from grey to white shown in the inset, indicating surface lattice defects transferred from surface to bulk or were oxidized by air.

**Figure 3:**
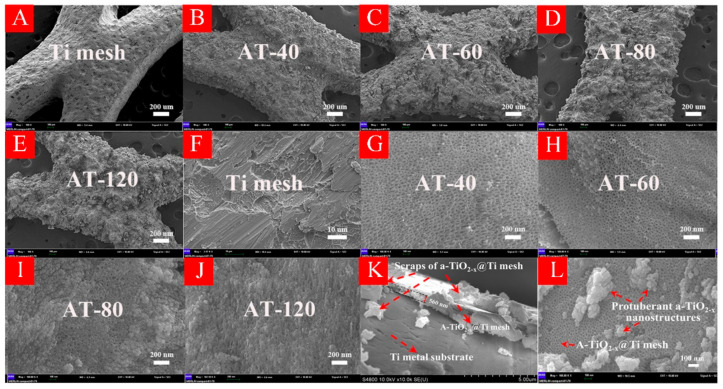
(**A**–**L**) display scanning electron microscopy (SEM) images of all HA-TiO_2−x_ samples. (**K**) shows the cross-section SEM image of AT-60. (**L**) shows some protuberances generated on Ti mesh surface.

In order to characterize the surface morphology in HA-TiO_2−x_, SEM examinations are illustrated in Figure 3. As shown in low-magnification SEM pictures from Figure 3A–E), all samples have rough surface compared with Ti mesh, and the surface corrosion increased with treatment time. As exhibited in high-magnification SEM pictures from Figure 3F–J, large quantities of nanopores with around 20 nm diameter arose on the Ti mesh surface as seen in AT-40 and AT-60. Observably, owing to the rough surface in AT-60 sample, some protuberances were formed on the nanopores surface where the thickness was about 560 nm as seen in Figure 3K,L. As seen in AT-80 and AT-120, these nanopores gradually vanished with the increase of the treatment time, which suggested that an appropriate anodization treatment time was necessary for fabrication of nanopores structures. As a result, amorphous TiO_2_ with massive nanopores structures was manufactured on the Ti mesh surface. On the other hand, these nano-structures also explained the excellent visible light absorption, because incident light can be diffused by the nanopores array which largely weakened the reflected light intensity [30]. The HRTEM images of AT-60 sample are shown in Figure S1.

### 3.2. Formation of O_V_ in HA-TiO_2−x_

**Figure 4:**
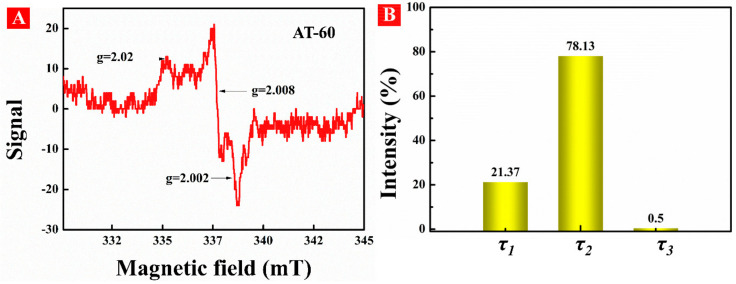
(**A**) EPR spectra and (**B**) positron lifetime and relative intensities of AT-60 sample.

Electron paramagnetic resonance (EPR) was conducted to confirm the existence of surface defects in HA-TiO_2−x_ as shown in Figure 4A. The EPR spectrum displayed apparent signals of g = 2.002, g = 2.008, and g = 2.02 which was performed at room temperature without light irradiation. The g-value of 2.002 is attributed to surface oxygen vacancies due to unpaired electrons trapped at the oxygen vacancies on TiO_2_ [31]. The signal of g = 2.008 is related to oxygen vacancies with one electron located in the sub-surface or bulk regions of TiO_2_ [32]. The signal of g = 2.02 is ascribed to O_2_^−^, which was generated from the reduction of adsorbed O_2_ by surface Ti^3+^ and thus confirmed the existence of surface Ti^3+^ [33]. Generally, the formation of O_V_ is always connected with the generation of Ti^3+^ species, and O_V_ has a strong ability to preserve the surface Ti^3+^ [34]. However, surface Ti^3+^ species are unstable due to the ambient oxidation and, therefore, the stability of surface O_V_ should be explained.

Positron annihilation spectrometry as a useful technology can characterize the size, type, and relative concentration of vacancies in the surface region of nanomaterial. As shown in Figure 4B, three kinds of positron lifetime components in AT-60 are referred to as τ_1_, τ_2_, τ_3_, with relative intensities noted as I_1_, I_2_, I_3_, respectively. The longest component (τ_3_) is generally considered as the annihilation of ortho-positronium atoms generated in large voids [35]. The smaller lifetime component (τ_2_) can be attributed to larger size defects for instance O_V_ clusters or surface defects [36]. The shortest component (τ_1_) resulted from free annihilation of the positrons in the lattice and at small O_V_ sites [37]. Herein, these results confirmed that the O_V_ defects not only distributed in the surface and subsurface, but also in interior regions of HA-TiO_2−x_, instead of merely accumulating on its surface. Compared with surface O_V_, subsurface or interior O_V_ can prevent gradual oxidation by air and water, and herein are quite stable.

**Figure 5:**
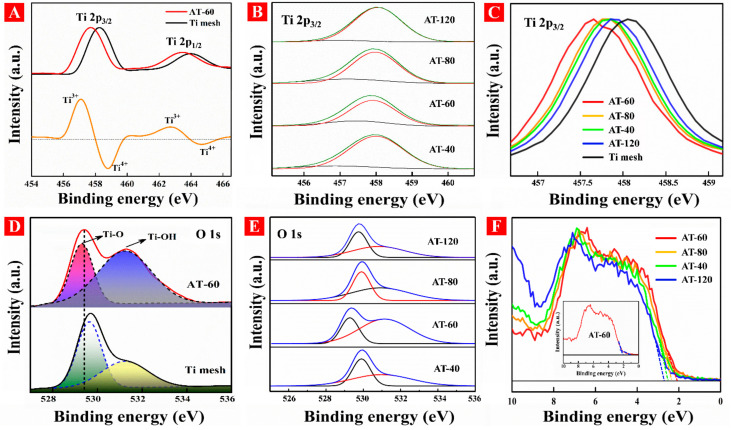
(**A**) Ti 2p spectra of AT-60 and Ti mesh. (**B**) Ti 2p_3/2_ spectra and (**C**) its enlarged view for all samples. (**D**) O 1s spectra of AT-60 and Ti mesh, and (**E**) the deconvoluted O 1s for all samples. (**F**) the valence band state spectra for all samples.

We further investigated surface chemical compositions and valence states for all samples by using XPS spectra as shown in Figure 5. All XPS spectra were calibrated with reference to C 1 s at 284.8 eV. No apparent differences among these samples are shown in full XPS spectra (Figure S2). From Figure 5A, the binding energies of Ti mesh located at 458.2 and 463.9 eV are ascribed to Ti 2p_3/2_ and Ti 2p_1/2_ peaks of Ti^4+^, respectively [38]. In comparison, AT-60 displayed an apparent negative shift in binding energy, which suggested Ti^3+^ species existed in the surface of HA-TiO_2−x_. We thus subtracted the Ti 2p spectra of AT-60 with that of Ti mesh as seen in the bottom of Figure 5A, exhibiting two distinguished peaks located at 457.1 and 462.8 eV that can be ascribed to Ti 2p_3/2_ and Ti 2p_1/2_ peaks of Ti^3+^, respectively [39]. The deconvoluted Ti 2p_3/2_ spectra in Figure 5B presented the Ti^3+^ and Ti^4+^ content variations along with the treatment time, and detailed data are listed in Table 1. Figure 5C demonstrates that binding energy shift of Ti 2p_3/2_ peak for all samples and AT-60 received the largest blue shift in binding energy with 457.8 eV. As displayed in Figure 5D, the deconvoluted peaks of O 1s of Ti mesh located at 529.3 and 531.1 eV can be attributed to the lattice oxygen (Ti-O) and surface absorbed oxygen (Ti-OH), respectively [40]. By comparison, the peak intensity of Ti-OH in AT-60 was much stronger than that of Ti mesh, indicating a higher concentration of hydroxyl groups in HA-TiO_2−x_. Moreover, a clear negative shift in binding energy of Ti-OH peak was due to the rich amount of surface O_V_, which can accumulate sufficient electrons [41]. Figure 5E exhibited the differences of deconvoluted O 1s spectra, in which AT-60 showed the strongest intensity of the Ti-OH peak. Figure 5F illustrated the shift of valence band spectrum with the increase of treatment time, and the valence band maximum (VBM) of AT-60 possessed the largest blueshift about 2.03 eV. The band tail of AT-60 was estimated about 1.27 eV as shown in inset of Figure 5F. The whole parameters including bandgap, VBM, and relative ratio of Ti^3+^/Ti^4+^ were shown in Table 1. With the increase of Ti^3+^ content (AT-40 to AT-60), both bandgap and VBM decreased, but when the decrease of Ti^3+^ content (AT-60 to AT-120), both bandgap and VBM increased. Herein, these results revealed Ti^3+^ content can directly engineer the electrical bandgap structure in HA-TiO_2−x_. The regulated mechanism of Ti^3+^ content should be scrutinized later.

### 3.3. Electronic Structures of HA-TiO_2−x_

**Figure 6:**
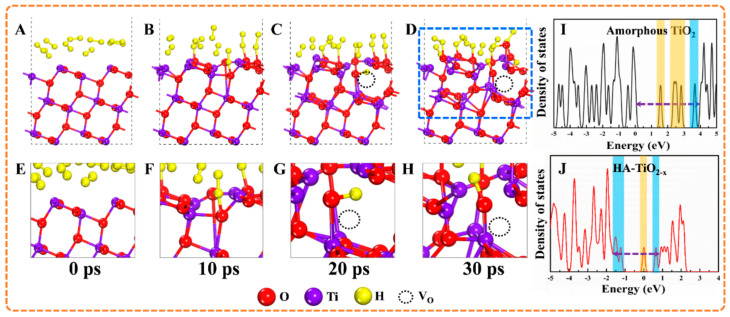
(**A**–**D**) are the simulated generation process of surface and subsurface O_V_ by density functional theory (DFT), and (**E**–**H**) are the corresponding enlarged views of the top surface. (**I**,**J**) are the electrical bandgap structures of amorphous TiO_2_ and HA-TiO_2−x_, respectively.

DFT calculation was employed to explore the surface O_V_ generation and the theoretical bandgap in HA-TiO_2−x_. As shown in Figure 6A–D, simulated time-resolved hydrogenation process of HA-TiO_2−x_ was illustrated with picosecond-scale frame. Originally, hydrogen atoms moved to amorphous TiO_2_ surface but without interface interaction shown in Figure 6A,E, and this moment was set as 0 ps. As time increased to 10 ps, several hydrogen atoms started bonding with surface oxygen atoms to generate Ti-OH bonds as shown Figure 6B,F. As time proceeded to 20 ps shown in Figure 6C,G, massive hydrogen atoms bonded with surface oxygen atoms, facilitating breaking up of Ti–O bonds on the surfaces of amorphous TiO_2_, which was represented as (~Ti-O~) + H_2_ → (~Ti-H) + (~O-H) [42]. Therefore, until now, surface O_V_ had been created, leading to the disordered surface simultaneously. When time reached to 30 ps, hydrogen atoms moved to the inner atomic layer to break the surrounding Ti-O bonds, resulting in the formation of subsurface O_V_ in HA-TiO_2−x_. Eventually, a stabilized disordered surface marked with blue dashed square was established after hydrogenation of amorphous TiO_2_. Herein, unique configuration of disordered surface@amorphous core in HA-TiO_2−x_ was generated. The partial density of states (PDOS) for amorphous TiO_2_ and HA-TiO_2−x_ are shown in Figure 6I,J, respectively. The bandgap of amorphous TiO_2_ was estimated at 3.68 eV, with narrow shallow states (marked with blue rectangle) near the valence band edge and several deep midgap trap states (marked with yellow rectangle) shown in Figure 6I. Observably, after hydrogenation, the number of midgap trap states was decreased, and the shallow states near the valence band edge and conduction band edge emerged in HA-TiO_2−x_ displayed in Figure 6J. The simulated bandgap of HA-TiO_2−x_ was around 2.45 eV which was consistent with the experimental value.

**Figure 7:**
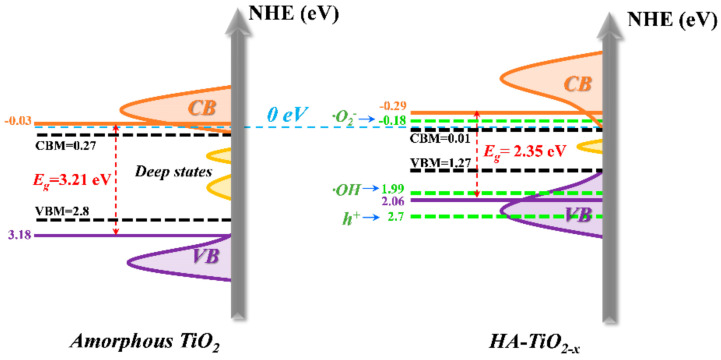
The schematic illustration of bandgap structure of amorphous TiO_2_ and HA-TiO_2−x_.

To clearly demonstrate the bandgap structures, the schematic illustration was presented in Figure 7.

The bandgap of amorphous TiO_2_ was measured from Figure S3 which was 3.21 eV and well accorded with theoretical value. As shown in Figure 7 (left), two continuous deep midgap trap states filled in the bandgap, which could have originated from large bulk voids or long-range lattice disorder in amorphous TiO_2_ [43]. As mentioned in previous literatures, these midgap trap states served as e-h recombination centers can inhibit the transition of electrons from valence band to conduction band (VB-CB for UV response), as well as from valence band to defect states (VB-defect for UV and visible light response) [13]. As for defect-CB (visible light response), the potential of superoxide radicals O_2_^−^ was lower than the conduction band maximum (CBM) of amorphous TiO_2_. Thus, photoinduced holes and electrons were wasted regardless of UV or longer wavelength light, which could explain the non-photoactive of amorphous TiO_2_. By contrast, unique configuration of disordered surface with an amorphous core in HA-TiO_2−x_ revealed distinct electrical bandgap framework compared with amorphous TiO_2_. Both band tail states (shallow states) near valence band and conduction band were generated, and the bandgap between VBM and CBM was estimated to 1.26 eV, resulting in extraordinary visible light absorption proved by DRS data. Therefore, to gain efficient light absorption, it is indispensable to broaden shallow states and narrow deep ones. Afterwards, in retrospect, the controversy with the origins of low-energy photons absorption and bandgap structure regulation theory in hydrogenated TiO_2−x_ nanomaterials should be discussed. First of all, our results proved that O_V_ disordered surface induced a narrow bandgap by introduced shallow states which was responsible for the low-energy photon absorption. Regarding hydrogenated crystalline TiO_2−x_ (disordered surface@crystalline core), we thus inferred that surface O_V_ could take the major role in visible light absorption (VB-Defect and/or Defect-CB), whereas the untreated inner-bulk crystalline region should be in charge of UV photons capturing (VB-CB). The annealed amorphous TiO_2_, i.e., crystalized anatase TiO_2_ shown in Figure 2D, was confirmed without surface O_V_ and associated visible-light photoactivity but has UV-responded photoactivity (Figures S4 and S5). However, the effect of crystalline core cannot be ignored yet as it can also modify bandgap by constructing the disorder/crystalline interface [17,18]. Moreover, the electric potentials of governing radicals in photoactivity including superoxide radical (−0.18 eV) and hydroxyl radical (1.99 eV) were posited in the bandgap of HA-TiO_2−x_. Nevertheless, the electric potential of h^+^ (2.7 eV) was much higher than that of VBM, as well as, the deep states below CBM acted as e-h pairs recombination centers, which could reduce the UV photoactivity.

### 3.4. Photocatalytic Performance Examinations

**Figure 8:**
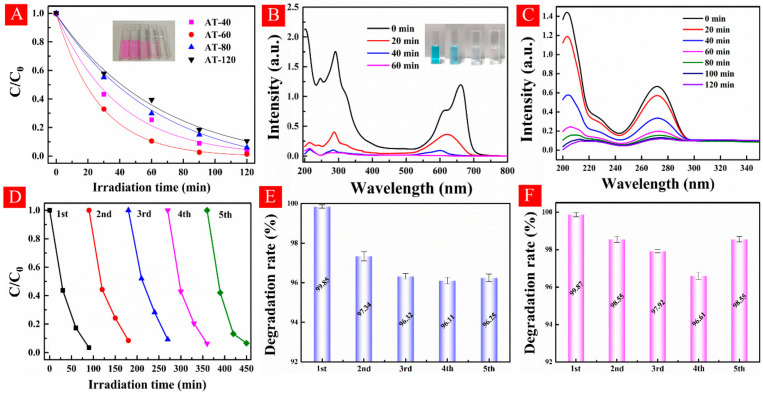
(**A**–**C**) are the visible-light photodegradation of RhB, MB, and theophylline, respectively. (**D**–**F**) are the recycling experiments of RhB, MB, and theophylline, respectively.

The photodegradation of rhodamine B under visible light illumination (λ > 420 nm) is shown in Figure 8A. AT-60 exhibited the best visible-light photoactivity in all samples. To evaluate its stability, repeated experiments for five times under the same conditions showed no obvious difference between each cycle in Figure 8D. The visible-light degradations of methyl blue and theophylline are shown in Figure 8B,C. Almost complete photodegradation of MB (99.85%) was obtained after 1 h visible light irradiation. Recycle experiments were also tested five times and it showed a stable performance in Figure 8E. Theophylline as a typical pharmaceuticals and personal care products (PPCPs) that has been universally applied in pharmaceuticals, food additives, and personal care products [44]. Nevertheless, insufficient decomposition of PPCPs in wastewater treatments enables the remains to disrupt human endocrines. As shown in Figure 8C, the intense peak located at 271.6 nm was regarded as the characteristic peak of theophylline. After a 2 h reaction, all peaks in ultraviolet range hugely attenuated and the photodegradation rate reached at 99.87%. Repeatability test was also conducted and exhibited a steady performance from Figure 8F. Finally, some representative studies about TiO_2_ nano-structures with high performance in dye photodegradation were provided for comparison with as-prepared HA-TiO_2−x_ as shown in Table S1, showing a much higher visible light photoactivity of HA-TiO_2−x_. In general, according to the above results, it can be concluded that visible-light photocatalytic efficiency was mainly affected by the optical bandgap of HA-TiO_2−x_.

**Figure 9:**
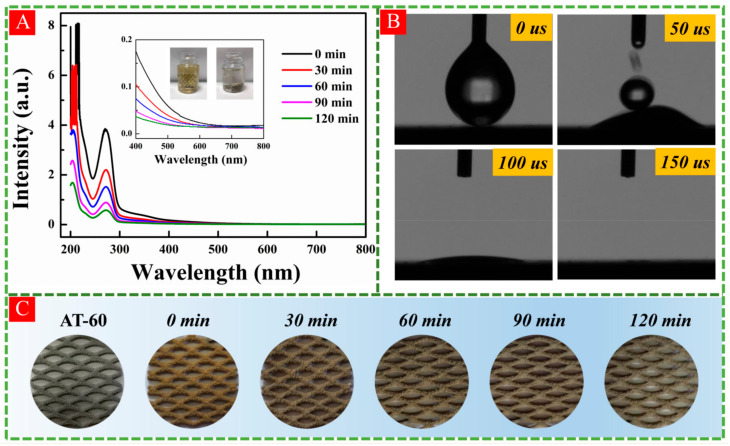
(**A**) Photocatalytic degradation curves of black tea water pollutants with 2 pieces AT-60 under visible-light irradiation, and the photographs of variations in black tea water as shown in inset. (**B**) The examinations of surface hydrophilic performance, and (**C**) the self-cleaning surface with degradation of absorbed organics in black tea water using AT-60.

To investigate the photocatalytic performance in actual pollution water, black tea water was applied in visible-light photodegradation experiment. Being rich in tea polyphenols, theaflavins, thearubigins, amino acids, and especially theophylline, black tea possesses many benefits for instance anti-cancer, anti-oxidant, anti-obesity, and atherosclerosis prevention [45]. From Figure 9A, the observed DRS curves of black tea water were similar with that of theophylline, which suggested that the main ingredient in black tea water was theophylline. Owing to the high concentration of black tea water, it showed several extremely sharp peaks in 200–220 nm range. During visible-light degradation, two pieces of AT-60 meshes with same size (2 × 2 × 1 cm^3^) were stacked in polyethylene plastic bottle filled with 50 mL black tea water. With the increase of irradiation time, the intensities of all peaks in ultraviolet region gradually decrease. Clearly, the color was changed from brown to almost transparent, responding to the decline of DRS curves in visible regions (400–600 nm) shown in the inset of Figure 9A. The photodegradation efficiency reached up to 85% over 2 h calculated by the peak intensity located at 271.6 nm. On the other hand, the super-hydrophilic surface of AT-60 was observed in Figure 9B, which led to a uniform brown color of black tea covered on AT-60 as seen in Figure 9C. Observably, the surface color varied from brown to grey again along with irradiation time in air, indicating the self-cleaning performance of HA-TiO_2−x_ surface.

**Figure 10:**
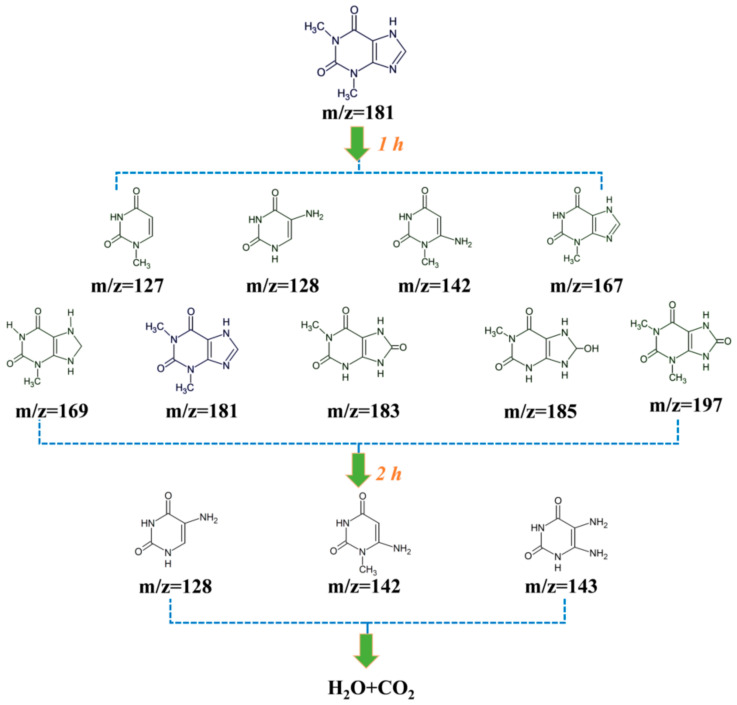
The possible visible light photodegradation pathway of theophylline using AT-60.

To investigate the visible-light photodegradation pathway of theophylline, the GC/MS system was used to analyse the intermediates as shown in Figure 10. Initially, only theophylline m/z = 181 was observed. After 1 h photodegradation, nine kinds of intermediates were detected and the detailed information was displayed in Table S2. The main compounds were theophylline m/z = 181, and 8-Hydroxy-1/3-methyl-3,7,8,9-tetrahydro-1H-purine-2,6-dione m/z = 185. After 2 h reaction, three kinds of intermediates were still observed, except theophylline. The relative content with treatment time is shown in Figure S6, which confirms the nearly total photodegradation of theophylline into CO_2_ and H_2_O.

### 3.5. Reactive Species Tests and Photodegradation Mechanism

**Figure 11:**
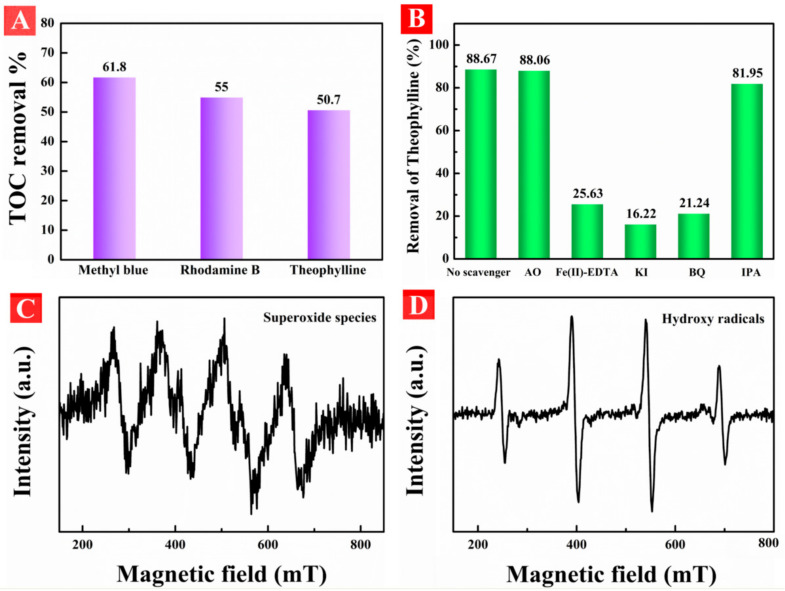
(**A**) The total organic carbon (TOC) tests for decomposed polluted water of RhB, MB, and theophylline. (**B**) reactive oxidant scavenging experiments. (**C**,**D**) were the electron paramagnetic resonance (EPR) detection of O_2_^−^ and OH, respectively, in aqueous solutions with visible light irradiation.

**Figure 12:**
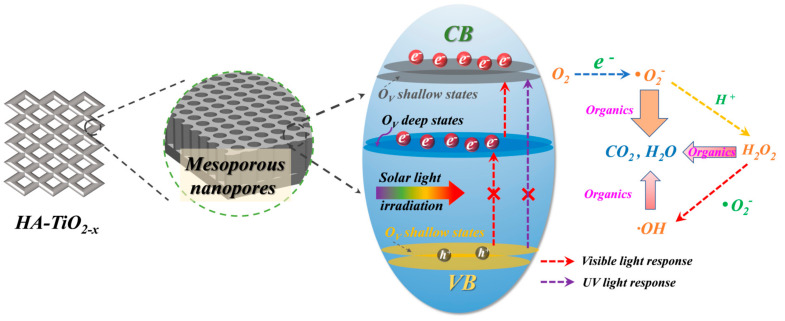
Proposed schematic diagram of visible–light photoactivity for HA-TiO_2−x_.

The total organic carbon (TOC) as an important evaluation for polluted water purification is shown in Figure 11A. The TOC removal rates after 2 h reaction of MB, RhB, and theophylline were measured to 61.8%, 55%, and 50.7%, respectively, which indicated that HA-TiO_2−x_ exhibited high visible-light photodegradation performance for wastewater purification. During the photocatalytic reaction process, different kinds of reactive species including OH, photoinduced holes (h^+^), O_2_^−^ and H_2_O_2_ are involved in degradation. To clarify the contribution, reactive species trapping experiments were carried out in the presence of AT-60 under visible-light irradiation. Five kinds of scavenger including ammonium oxalate (AO, h^+^ scavenger), Fe(II)-EDTA (H_2_O_2_ scavenger), potassium iodide (KI, OH_ads_ and electron scavenger), *p*-benzoquinone (BQ, O_2_^−^ scavenger), and isopropanol (IPA, scavenger for OH in the bulk solution) were applied in photodegradation. As shown in Figure 11B, the visible-light photodegradation rate of AT-60 without scavenger was 88.67%, while in the presence of AO, Fe(II)-EDTA, KI, BQ, and IPA were 88.06%, 25.63%, 16.22%, 21.24%, and 81.95%, respectively. Therefore, Fe(II)-EDTA, KI, and BQ can heavily hinder theophylline photodegradation performance but AO and IPA had no influence. Evidently, O_2_^−^, H_2_O_2_, and OH_ads_ (adsorbed OH radicals on catalyst surface) were the dominant reactive species contributing to high visible-light photoactivity in HA-TiO_2−x_. Moreover, the similar situations were observed with RhB and MB in presence of above scavengers using AT-60, as shown Figure S7. Moreover, EPR signals of O_2_^−^ and OH were verified by applying in-situ trap the spin-reactive species as shown in Figure 11C,D. Moreover, the amount of O_2_^−^ was 2.7 times as much as that of OH as listed in Table S3, suggesting O_2_^−^ principally contributed to the high visible light photodegradation. In conclusion, solid evidence confirmed that O_2_^−^ and OH accounted for visible light photodegradation, however, h^+^ did not participate in the visible photoactivity. To further confirm the effect of h^+^, UV-light photodegradation of HA-TiO_2−x_ was carried out. As shown in Figure S8, the poor photoactivities using the AT-60 sample indicated UV light responded transitions of VB-CB and/or VB-defect state were almost invalid. On the basis of the above experimental results and theoretical analyses, schematic diagram of the photodegradation of HA-TiO_2−x_ are illustrated in Figure 12.

### 3.6. Verification of Long-Term Stability of Surface O_V_

**Figure 13:**
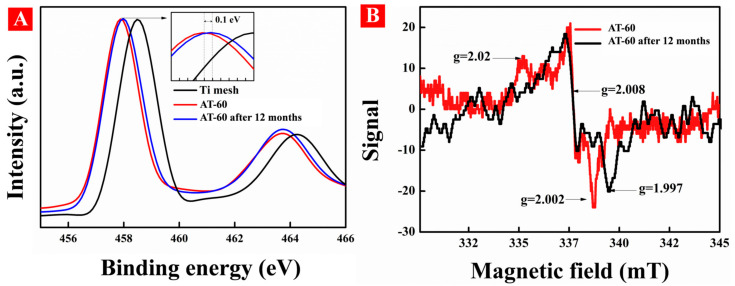
(**A**) X-ray photoelectron spectra (XPS) and (**B**) EPR spectra of the AT-60 and it after 12 months storages in ambient.

In order to confirm the stability of surface O_V_ in HA-TiO_2−x_, the AT-60 sample after 12 months storage was used to conduct XPS and EPR examinations. As in Figure 13A, there was a little positive shift in binding energy after photodegradation usage, but it still showed an obvious Ti^3+^ peaks according to XPS results, suggesting the existence of surface O_V_ and Ti^3+^ species. The EPR spectrum of AT-60 after 12 months storage showed two kinds of signal of subsurface O_V_ (g = 2.008) and bulk Ti^3+^ species (g = 1.997) [46]. Unfortunately, the surface Ti^3+^ species (g = 2.02) and surface O_V_ (g = 2.002) disappeared as shown in Figure 13B. In expectation, O_V_ and Ti^3+^ species at the surface layer of HA-TiO_2−x_ were all recovered, but unexpectedly, interior defects were still preserved, leading to high visible photodegradation again (Figure S9). Anyhow, mere interior defect structure cannot explain the stubborn subsurface O_V_, especially in the presence of strong liquid plasma oxidation. Herein, the stability of subsurface O_V_ in HA-TiO_2−x_ should be further clarified.

### 3.7. The Formation Mechanism of HA-TiO_2−x_

Given the above results and analyses, the formation mechanism of disordered surface with the amorphous core structure should be discussed. The essence of liquid plasma-induced hydrogenation in our case is considered identical with thermal hydrogenation. According to current experience in thermal hydrogenation, the longer hydrogenation proceeds, the higher the concentration of surface Ti^3+^ species, and the more visible-light photons hydrogenated TiO_2−x_ absorbs [3]. Nevertheless, it was confusing that visible-light absorption in HA-TiO_2−x_ represented a completely opposite trend that weakened the visible-light response achieved if the treatment time was prolonged (according to DRS results). Actually, apart from the hydrogenation reaction, liquid plasma also generated many kinds of active substances including electrons, hydroxyl radical, hydrogen peroxide, and ultraviolet radiation, which enables strong oxygenation with as-prepared HA-TiO_2−x_. As a result, there existed two intense reactions associated with liquid plasma-induced hydrogenation and oxidation. In the primary stage, hydrogenation played the major effect, leading to O_V_ disordered surface with treatment time (AT-40 and AT-60). Oxidation took a small effect owing to the low concentration of active substances during the primary status of liquid plasma generation [47]. When the treatment time increased and reached a threshold, the oxidation effect dominated the whole reactions and set to heal the surface O_V_. In addition, anodization of Ti mesh anode can accelerate the surface corrosion and surface amorphization, resulting in oxygenation of surface O_V_ (AT-80 and AT-120). The bandgap engineering in HA-TiO_2−x_ can be manipulated through controlling the synergy effect of hydrogenation and oxidation in liquid plasma, and especially, regulation of the synergistic treatment time. Finally, some analyses should be undertaken with respect to the stability of subsurface O_V_. On the one hand, the as-obtained surface amorphization by anodization can produce the top amorphous layer wrapped on HA-TiO_2−x_, hindering further oxidation. On the other hand, these O_V_ and Ti^3+^ species at a disordered surface could form point defect structure of Ti^3+^-O_V_-Ti^3+^, which was verified rather stable because of the electrostatic balance [48]. Accordingly, both surface amorphization and inner-bulk defect structure prohibited liquid plasma from recovering interior O_V_, which explained the long-term stability in the harsh environment. The schematic representation of the formation mechanism was shown in Figure S10. We also tried virous discharge times such as 20, 40, 60, 80, 100, 120, and 150 min, and the results of all samples including XRD, DRS, and visible-light photodegradation are shown in Figure S11 and Table S4.

## 4. Conclusions

In summary, hydrogenated amorphous TiO_2−x_ (HA-TiO_2−x_) with stable surface O_V_ has been successfully prepared. The highlights and novelties in this work are as below.

1. Hydrogenated amorphous TiO_2−x_ was reported for the first time. First-principle calculations revealed the unique bandgap structure that both band tail states near valence band and conduction one were generated, leading to extraordinary visible-light absorption.
2. The distinct liquid plasma hydrogenation strategy can effectively produce abundant surface O_V_ on amorphous TiO_2_.
3. The special photodegradation mechanism. In visible-light photodegradation, O_2_^−^ and OH were accounted for polluted water decomposition, nevertheless, h^+^ was almost not contributed to the visible photoactivity.
4. The concentration of O_V_ heavily affected photocatalytic efficiency. The higher O_V_ concentration the HA-TiO_2−x_ possessed, the narrower the bandgap it received, and the higher photocatalytic efficiency it exhibited.
5. The excellent visible-light photodegradation and stability. HA-TiO_2−x_ exhibited superior visible-light photodegradations in RhB (98.7%), MB (99.85%), and theophylline (99.87). Moreover, surface O_V_ in HA-TiO_2−x_ was rather stable and can be preserved in an ambient atmosphere over 12 months.

This study provided a novel type of hydrogenated TiO_2−x_ photocatalyst, which could trigger a series of visible light-driven amorphous photocatalysts in practical solar light conversion.

## Figures and Tables

**Table 1:** The parameters including bandgap, valence band maximum (VBM), and relative ratio of Ti^3+^/Ti^4+^.

Sample	Bandgap (eV)	Mean ± SD (eV)	VBM (eV)	Mean ± SD (eV)	Ti^3+^/Ti^4+^ (%)
AT-40	2.57	2.57 ± 0.03	2.38	2.38 ± 0.02	16%
AT-60	2.35	2.35 ± 0.01	2.03	2.03 ± 0.03	26%
AT-80	2.52	2.52 ± 0.02	2.24	2.24 ± 0.01	22%
AT-120	2.66	2.66 ± 0.01	2.55	2.55 ± 0.02	13%

Notes: SD = standard deviation.

## Data Availability

The data presented in this study are available on request from the corresponding author. The data are not publicly available due to the reason that the data also forms part of an ongoing study.

## Supplementary Materials

The following are available online at https://www.mdpi.com/article/10.3390/nano11112801/s1, Figure S1: (A) and (B) are the high resolution transmission electron microscopy (HRTEM) images of AT-60 sample, and (C) is the energy dispersive X-ray spectrometry (EDS) spectrum of green rectangle marked region in Figure S1(B). Figure S2: The full XPS spectra for all HA-TiO_2-__x_ samples. Figure S3: The DRS and valence band spectra of amorphous TiO_2_ nanopowder, the band tail state is posited at 2.8 eV (red line). Figure S4: The EPR spectra of anatase TiO_2_@Ti mesh and AT-60. Figure S5: The photoactivity of anatase TiO_2_@Ti mesh under UV light irradiation for 1 h. Figure S6: The relative content of intermediate products with irradiated time during visible-light photodegradation of theophylline using AT-60. Figure S7: The reactive oxidant scavenging experiments of (A) MB and (B) RhB using AT-60. Figure S8: The UV photodegradation experiments of RhB, MB, and theophylline using AT-60. Figure S9: The recycle test of AT-60 after 12 months storage under visible light illumination for 2 h. Figure S10: The schematic representation of the formation mechanism of HA-TiO_2−x_. (A) is the interaction process between hydrogen atoms and amorphous TiO_2_ surface, and (B) is the detailed formation process of HA-TiO_2−x_ nanopores. Figure S11: (A) XRD patterns and (B) DRS spectra of untreated Ti mesh and all samples. (C) the plots (αhν)^1/2^ versus hν by using the Kubelka-Munk function. (D) the rhodamine B photodegradation experiments under visible light for all samples. Table S1: Representative studies about TiO_2_ nano-structures with high performance in dye photodegradation for comparison with HA-TiO_2−x_@Ti mesh photocatalyst. Table S2: Main products during visible light degradation of theophylline. Table S3: The amount of ·O_2_^−^ and ·OH by employing of 5,5-dimethyl-1-pyrroline-*N*-oxide (DMPO) to in situ trap the spin-reactive species. Table S4: The bandgap of all samples. Supplementary Materials including some supplementary figures and tables [49,50,51,52,53,54,55,56,57,58,59].
