# Supplementary material for: Hydrogenated Amorphous TiO2−x and Its High Visible Light Photoactivity
Source: Nanomaterials (Basel). 2021 Oct 22;11(11):2801. doi: 10.3390/nano11112801 (PMC8625909; doi:10.3390/nano11112801)
Supplement: Supplementary file 1 [file nanomaterials-11-02801-s001.zip › nanomaterials-1412138-Supplementary.pdf]

Hydrogenated Amorphous TiO_{2-x} and Its High Visible Light Photoactivity

Guang Feng [1,2], Mengyun Hu [1,2,3], Shuai Yuan [1,2], Junyi Nan [3], and Heping Zeng [1,2,3,4,5,*]

1 Shanghai Key Laboratory of Modern Optical System, Engineering Research Center of Optical Instrument and System, Ministry of Education, School of Optical-Electrical and Computer Engineering, University of Shanghai for Science and Technology, Shanghai 200093, China; sunnyfeng1992@163.com (G.F.); myhu@phy.ecnu.edu.cn (M.H.); ye_zoom@126.com (S.Y.)
2 Chongqing Key Laboratory of Precision Optics, Chongqing Institute of East China Normal University, Chongqing 401120, China
3 State Key Laboratory of Precision Spectroscopy, East China Normal University, Shanghai 200062, China; nanjunyigood@163.com
4 CAS Center for Excellence in Ultra-Intense Laser Science, Shanghai 201800, China
5 Jinan Institute of Quantum Technology, Jinan 250101, China
* Correspondence: hpzeng@phy.ecnu.edu.cn

1. Results and discussion

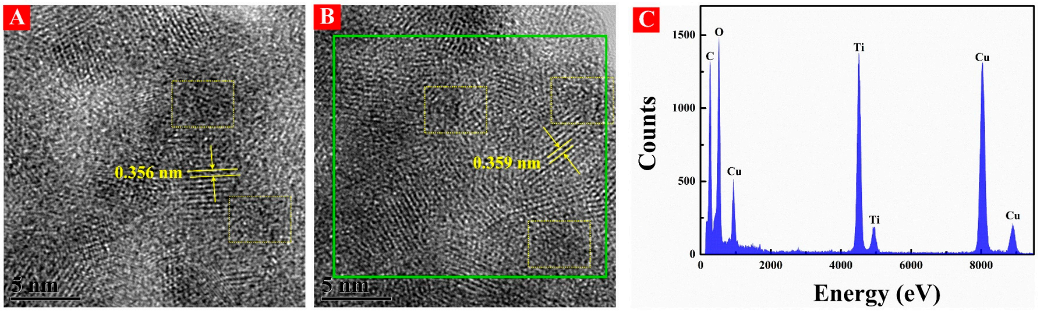

Figure S1. (A) and (B) are the high resolution transmission electron microscopy (HRTEM) images of AT-60 sample, and (C) is the energy dispersive X-ray spectrometry (EDS) spectrum of green rectangle marked region in Figure S1(B).

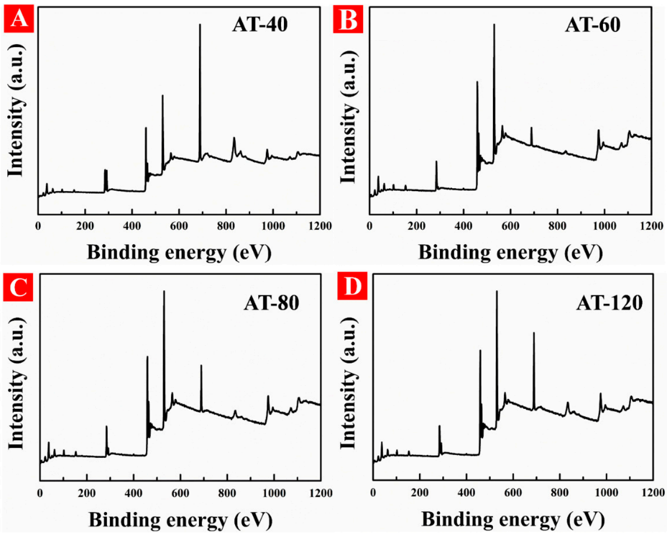

Figure S2. The full XPS spectra for all HA-TiO_{2-x} samples.

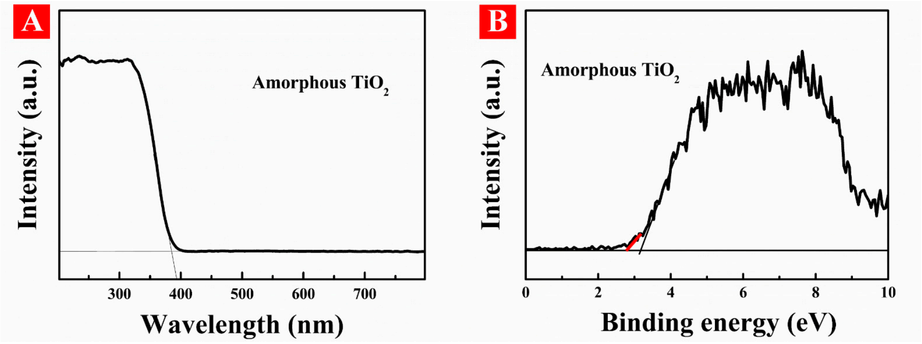

Figure S3. The DRS and valence band spectra of amorphous TiO_2 nanopowder, the band tail state is posited at 2.8 eV (red line).

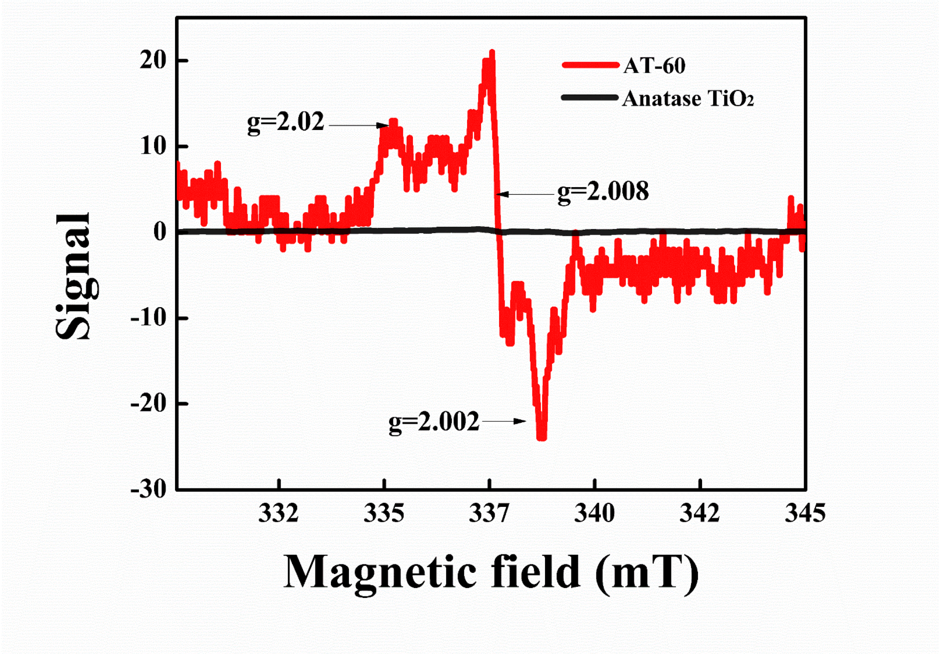

Figure S4. The EPR spectra of anatase TiO_2@Ti mesh and AT-60.

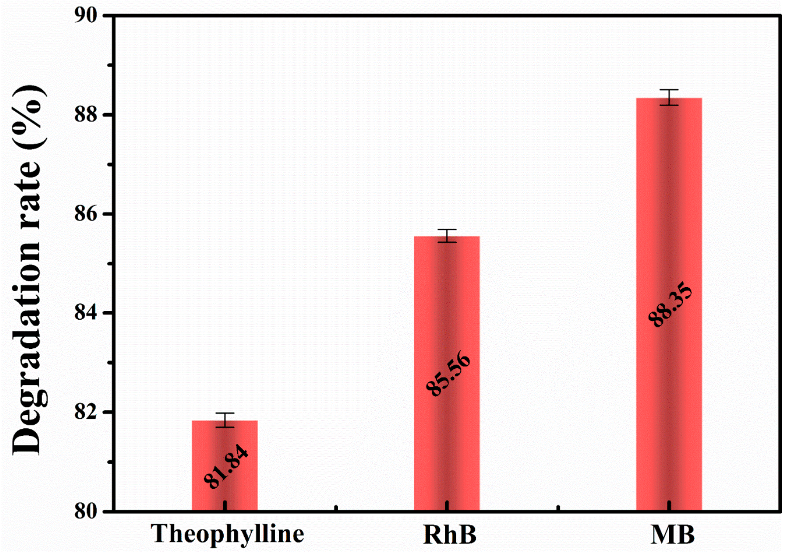

Figure S5. The photoactivity of anatase TiO_2@Ti mesh under UV light irradiation for 1 hour.

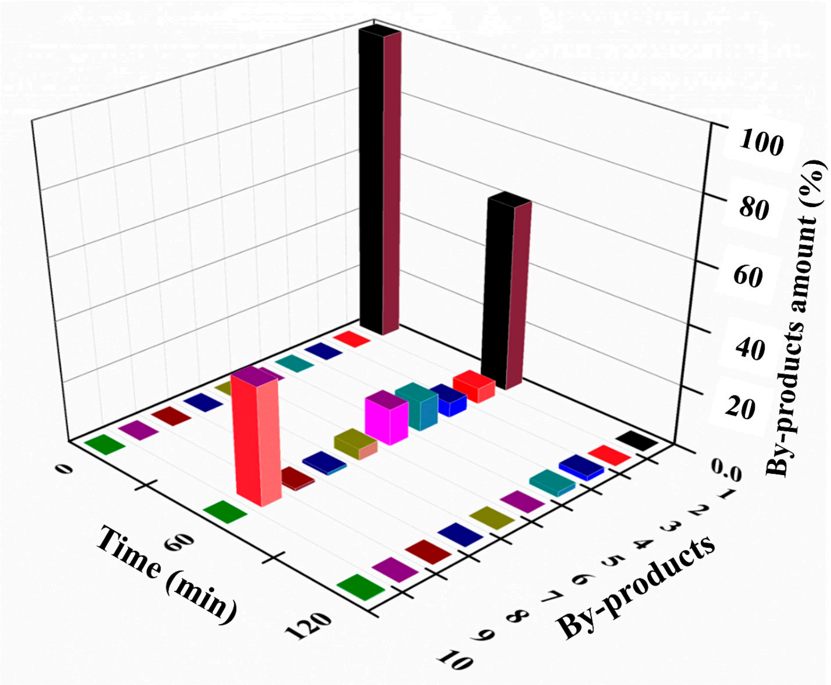

Figure S6. The relative content of intermediate products with irradiated time during visible-light photodegradation of theophylline using AT-60.

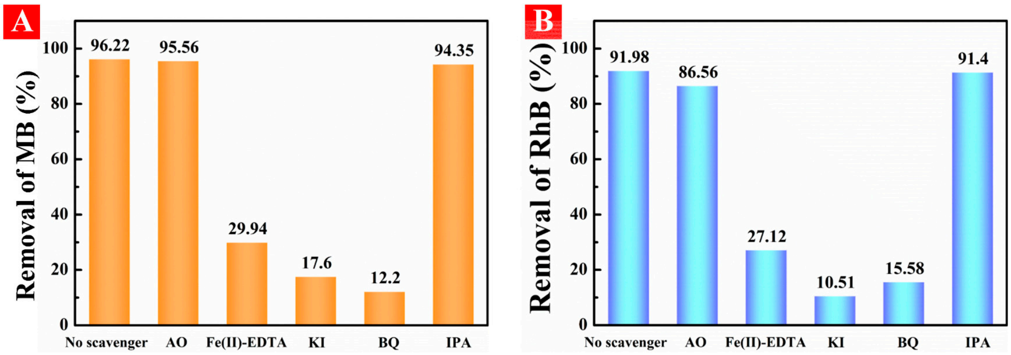

Figure S7. The reactive oxidant scavenging experiments of (A) MB and (B) RhB using AT-60.

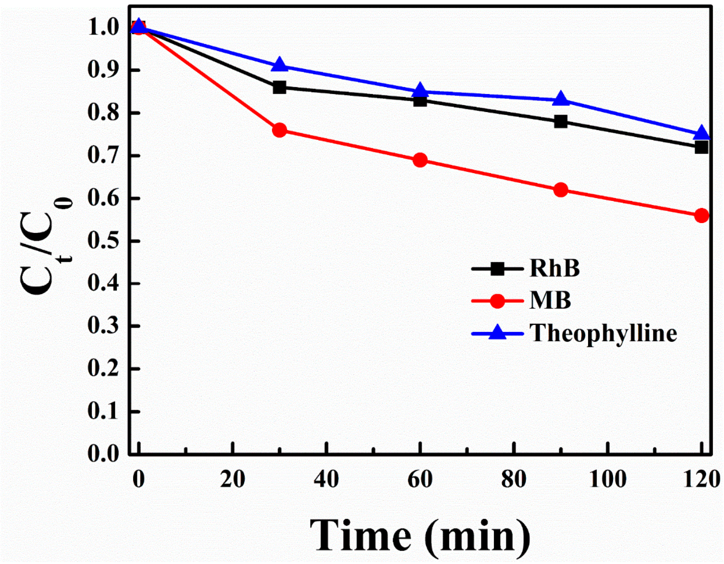

Figure S8. The UV photodegradation experiments of RhB, MB, and theophylline using AT-60.

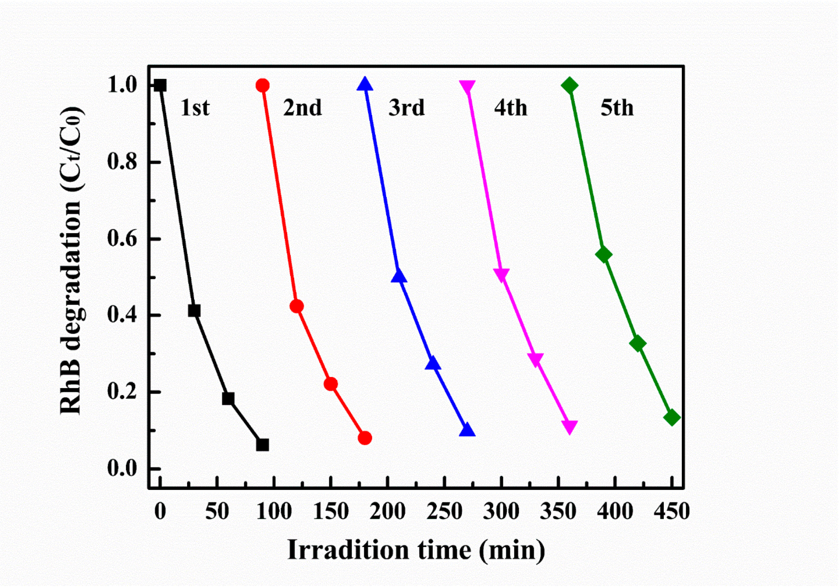

Figure S9. The recycle test of AT-60 after 12 months storage under visible light illumination for 2 h.

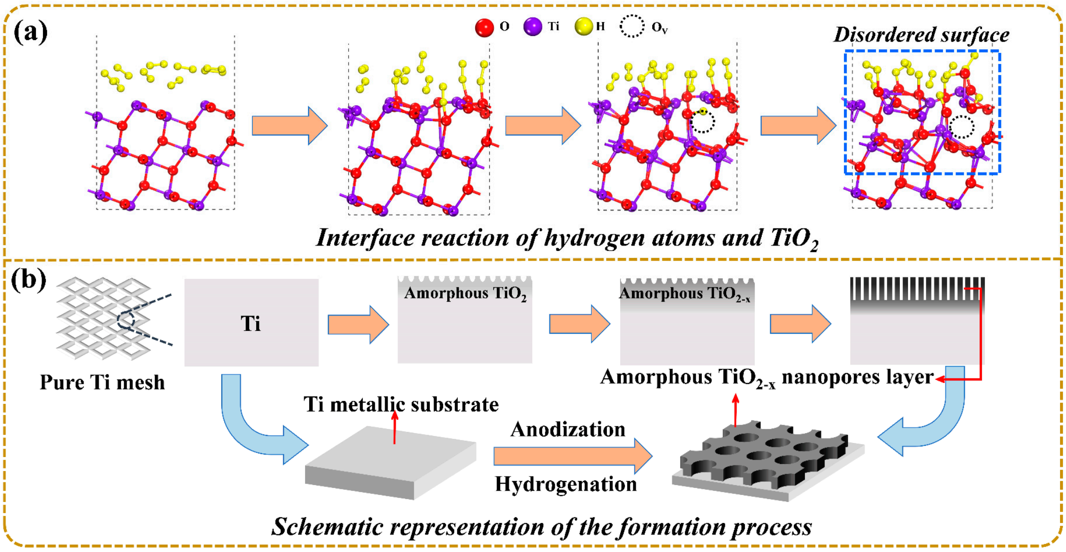

Figure S10. The schematic representation of the formation mechanism of HA-TiO_{2-x}. (A) is the interaction process between hydrogen atoms and amorphous TiO_2 surface, and (B) is the detailed formation process of HA-TiO_{2-x} nanopores.

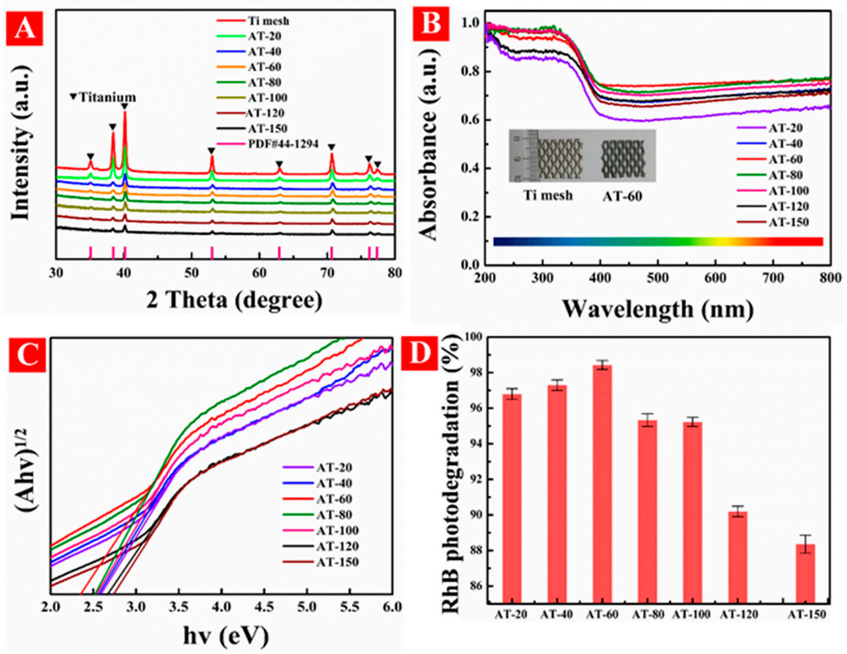

Figure S11. (A) XRD patterns and (B) DRS spectra of untreated Ti mesh and all samples. (C) the plots $(\alpha h\nu)^{1/2}$ versus hν by using the Kubelka-Munk function. (D) the rhodamine B photodegradation experiments under visible light for all samples.

Table S1. Representative studies about TiO_2 nano-structures with high performance in dye photodegradation for comparison with HA-TiO_{2-x}@Ti mesh photocatalyst.

Photocatalyst	Synthesis method	Light Source	Concentration	Weight	Performance	Ref.
HA-TiO_{2-x}@Ti mesh	Liquid plasma hydrogenation and anodization	Visible light, 300 W	50 ml MB, 10 mg/L	4 cm^2	$T_{90\%}$=40 min	This work
Defective TiO_{2-x} on Ti foil	Two-step anodization	Blue light, (400–500 nm)	6 mL RhB, 2 mg/L	4 cm^2	$T_{90\%}$>4 h	[49]
Ti mesh supported WO_3/TiO_2 nanotubes	Anodic oxidation and electrodeposition methods	Visible light (λ>400 nm), 300 W	50 ml MB, 10 mg/L	8.75 cm^2	$T_{72\%}$=120 min	[50]
Graphitic carbon nitride nanosheets	Two steps of calcination	Simulated sunlight 300 W xenon lamp	5 mL RhB, 50 mg/L	1 cm^2	$T_{90\%}$=150 min	[51]
TiO_2/g-C_3N_4 @Ti substrate	Plasma electrolytic oxidation and hydrothermal deposition	LED visible light 5 W	20 mg/L RhB	1 cm^2	$T_{50\%}$=600 min	[52]

TiO_2/ZnO nanocomposite films	Electrochemical and chemical bath deposition methods	UVA light	3 ml RhB 5 mg/L	3.6 cm^2	$T_{50\%}$=210 min	[53]
In_2O_3/TiO_2 nanotube arrays hybrids	Anodization and solvothermal method	300 W Xe lamp with a 365 nm cut-off filter	3 mL RhB 10 mg/L	3 cm^2	$T_{80\%}$=150 min	[54]
Ag/ZnO@indium tin oxide	Radio frequency (RF) sputtering	500 W Xe lamp 400−780 nm	20 mL 20 ppm,RhB	-	$T_{80\%}$=120 min	[55]
Cu_xO@Cu nanocomposites @ Cu mesh	Spinach leaf extract	Sunlight irradiation of 7 mW/cm^2	30 mL 10 mg/L MB	4 cm^2	$T_{90\%}$=50 min	[56]
TiO_2 nanotube arrays	Anodization and calcination	365 nm, 1.08 mW/cm^2	3 mL MB, 10 ppm	64 mm^2	$T_{90\%}$=8 h	[57]
TiO_2 nanorods/nanosheets	Hydrothermal reaction and annealing	Xe lamp, 67 mW/cm^2	50 mL MB, 5 mg/L.	2 cm^2	$T_{73.56\%}$=150 min	[58]
Nanosheets of Bi_2MoO_6	Solvothermal method and annealing	Xe lamp, 500 W	MB, 2×10^{-5} mol/L	2 cm^2	$T_{100\%}$=100 min	[59]

Table S2. Main products during visible light degradation of theophylline.

Sl.no.	Product	Mol.wt.	Elemental composition	m/z	
1	1/3-Methylpyrimidine-2,4(1*H*,3*H*)-dione	126.11	$C_5H_6N_2O_2$	127	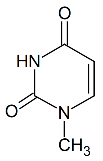
2	5/6-Aminopyrimidine-2,4(1*H*,3*H*)-dione	127.10	$C_4H_5N_3O_2$	128	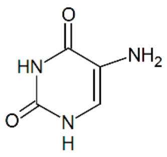
3	5/6-Aminoderivativeof1/3-methylpyrimidine-2,4(1*H*,3*H*)-dione	141.13	$C_5H_7N_3O_2$	142	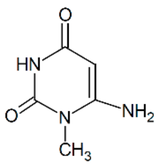

4	5,6-Diaminopyrimidine-2,4(1*H*,3*H*)-dione	142.12	$C_4H_6N_4O_2$	143	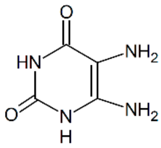
5	3-Methylxanthine	166.14	$C_6H_6N_4O_2$	167	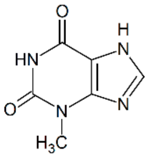
6	1/3-Methyltetrahydro-1*H*-purine-2,6-dione	168.15	$C_6H_8N_4O_2$	169	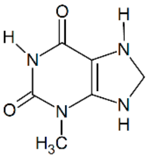
7	Theophylline	180.16	$C_7H_8N_4O_2$	181	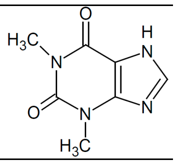
8	3-Methyluricacid	182.14	$C_6H_6N_4O_3$	183	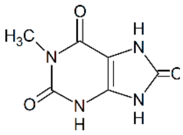
9	8-Hydroxy-1/3-methyl-3,7,8,9-tetrahydro-1*H*-purine-2,6-dione	184.15	$C_6H_8N_4O_3$	185	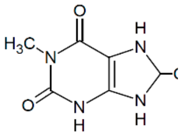
10	1,3-Dimethyluricacid	196.16	$C_7H_8N_4O_3$	197	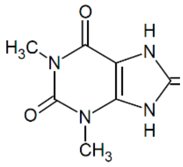

Table S3. The amount of $\cdot O_2^-$ and ·OH by employing of 5,5-dimethyl-1-pyrroline-*N*-oxide (DMPO) to in situ trap the spin-reactive species.

Sample	spins/mm^3	M	spins
$\cdot O_2^-$	7.686×10^{12}	1.276×10^{-5}	1.569×10^{14}
·OH	2.831×10^{12}	4.7×10^{-6}	6.02×10^{13}

Notes: "Spins/mm^3" is the number of spins per unit volume, "M" is the molarity, and "spins" is the total number of spins, and thus the free radicals can be estimated with spins/mm^3.

Table S4. The bandgap of all samples.

Sample	Bandgap (eV)	Mean±SD (eV)
**AT-20**	2.58	2.58±0.01

**AT-40**	2.57	2.57±0.03
**AT-60**	2.35	2.35±0.01
**AT-80**	2.52	2.52±0.02
**AT-100**	2.53	2.53±0.01
**AT-120**	2.66	2.66±0.01
**AT-150**	2.74	2.74±0.03

Notes: SD = standard deviation.
